# Moral Reasoning and Final-Year Undergraduate Dentistry Students in Australia: A Cross-Sectional Questionnaire Study

**DOI:** 10.3390/dj13110523

**Published:** 2025-11-07

**Authors:** Maurice J. Meade, Xiangqun Ju, David Hunter, Lisa Jamieson

**Affiliations:** 1 Orthodontic Unit, Adelaide Dental School, University of Adelaide, Adelaide, SA 5000, Australia; 2 Australian Research Centre for Population Oral Health, Adelaide Dental School, University of Adelaide, Adelaide, SA 5000, Australia; xiangqun.ju@adelaide.edu.au (X.J.); lisa.jamieson@adelaide.edu.au (L.J.); 3 Adelaide Faculty of Health and Medical Sciences, University of Adelaide, Adelaide, SA 5000, Australia; david.hunter@adelaide.edu.au

**Keywords:** moral reasoning, dental education, dental ethics

## Abstract

**Background/Objectives:** Proficiency in moral reasoning is essential for healthcare providers to successfully navigate ethically challenging decision-making. It is critical that student dentists about to enter practice have well-developed moral reasoning skills to ensure optimal patient care. The aim of the present study was to investigate the moral reasoning ability of students undertaking their final year of the undergraduate Bachelor of Dental Surgery (BDS) programme at the University of Adelaide (UofA) in Australia. **Methods**: Sixty-six final-year BDS students were invited to participate in an e-survey which included the Defined Issues Test 2 (DIT-2), a validated instrument for measuring moral reasoning. Calculated DIT-2 scores incorporatedthe postconventional (P) score and N2 score. Data analysis of demographic details and scores related to the DIT-2 included the use of t-tests, Mann–Whitney and the Spearman rank correlation coefficient tests. **Results**: A response rate of 45.5% (n = 30) was recorded. The mean (95% CI) P and N2 scores were 37.80 (32.04, 43.56) and 42.12 (37.72, 46.53), respectively. Scores for females and for those who had undertaken the majority of their pre-BDS studies outside of Australia and New Zealand were higher, but the differences were not statistically significant (*p* > 0.05). A Spearman Correlation Coefficient test indicated that age was moderately associated (r = 0.41; 95% CI: 0.01, 0.65; *p* = 0.04) with N2 scores. **Conclusions**: Moral reasoning scores were comparable to studies among similar cohorts conducted in other countries but were less than the scores considered optimal for a healthcare provider to proficiently manage challenges to ethical decision-making. Consideration should be given to the introduction of appropriate formal training in ethics to better manage these challenges.

## 1. Introduction

Adherence to a set of standards concerning professional conduct and behaviour is a requirement for initial and continued registration as a health practitioner with regulatory agencies internationally [1,2,3,4,5]. The objective of relevant dental agencies is to ensure the protection of the public, which is generally predicated on the principles of beneficence, non-maleficence, autonomy, fairness and veracity [3,5,6,7]. Additional objectives include the protection of the title of ‘dentist’ [2,3] and maintenance of confidence in the dental profession [2]. In Australia, students undertaking clinical training in healthcare are also required to be registered with the country’s health professional regulatory organisation—the Australian Healthcare Professional Regulation Agencyand must comply with the Agency’s Code of Conduct [3].

Agreement is lacking with regard to what constitutes professionalism in the context of dental registrant conduct [8,9,10,11,12]. However, some authors contend that compliance with sets of regulator standards may not be sufficient to guide registrants in acquiring solutions to specific ethical challenges, and that ethical behaviour corresponds to being able to successfully navigate ethical issues in addition to behaving professionally [10,13]. Bataglia and Bortolonza asserted that the ethical consideration of issues becomes more sophisticated when it incorporates the contemplation and reflection of rules and not just unquestioning acquiescence with them [14,15]. They also state that this is more likely to occur in the presence of well-developed moral reasoning skills [14].

Moral reasoning has been defined as the mental activity that an individual undertakes to examine moral challenges and arrive at potential answers [16]. It is a key factor in moral behaviour [17,18]. The Defining Issues Test 2 (DIT-2) is a commonly used tool in moral reasoning research [18,19,20]. It was developed to assess the psychological construct that marks the steps employed through which individuals decide that one mode of conduct in a specific circumstance is morally correct and another mode is morally wrong [21]. The test, which is derived from the original longer Defining Issues Test (DIT), has been determined to be reliable and valid and employs five stories incorporating moral dilemmas [20,22,23]. The test requires the respondent to rate and rank a variety of statements related to each story [20].

It appraises three lenses (schemas) that people employ in moral decision-making and aligns with the concept that moral development concerns changes that happen in an individual’s structure or form of thought [24]. The responses to the test can be evaluated to calculate a score that indicates the level to which an individual schema is adopted in moral decision-making [16]. Those individuals who adopt the Personal Interest (PI) schema in moral decision-making concentrate on how the decision would affect them rather than societal impact more broadly [16,18]. The decision-making of individuals who employ the Maintaining Norms (MN) schema are informed by the importance of acquiescence with rules and regulations and the upholding of social order [25]. Those whose moral decision-making is through a postconventional (*p*) schema are considered to have a more sophisticated approach compared with those who use the PI and MN decision-making strategies, as decisions are based on a deeper consideration of the issue rather than following traditional approaches to issues [16]. The N2 score is determined on the basis of whether items related to the P schema are chosen with higher priority than those concerning the PI or MN schemas [23,25].

Numerous studies have explored moral reasoning in individuals undertaking undergraduate studies in clinical disciplines including medicine [26,27], pharmacy [20,28,29,30,31], nursing [32,33] and dentistry [22,34,35,36,37]. Investigation, however, related to moral reasoning in undergraduate dentistry students in Australia is lacking. The findings from relevant research have the potential to inform students about their own thoughts and attitudes about ethical decision-making. They can also guide educators regarding the teaching of ethics during undergraduate dentistry programmes and provide the wider dental profession with information concerning the moral reasoning skillset of individuals who are about to enter practice as qualified dentists. The aim of the present study, therefore, was to investigate the moral reasoning scores of students undertaking their final year of the undergraduate Bachelor of Dental Surgery (BDS) programme at the University of Adelaide (UofA) in Australia.

## 2. Materials and Methods

The UofA Human Research Ethics Committee approved the present cross-sectional questionnaire survey (H-2024-082) which was part of a wider project regarding professionalism and moral reasoning in dental education in Australia. Informed consent to participate was obtained from all the participants in the study. It was designed and developed on the Qualtrics (Silver Lake and the Canada Pension Plan Investment Board, Seattle, DC, USA) software facility and aimed to conform with recommendations regarding optimal survey design and reporting [38,39]. The Strengthening the Reporting of Observational Studies in Epidemiology (STROBE) Statement checklist for cross-sectional studies was employed to optimise reporting of the present study [40].

### 2.1. Survey Instrument

The survey comprised two sections. Section 1 contained questions related to demographic details regarding respondent age, sex, country in which most of the respondent education was undertaken prior to their BDS studies and whether English was the respondent’s first language.

Section 2 consisted of the five ethical dilemmas contained within the DIT-2 [21]:

I. A parent deliberating the theft of food for his hungry family.
II. A reporter considering publication of a negative article concerning a politician campaigning for election.
III. A chair of a school committee determining whether to cancel a controversial gathering.
IV. A healthcare provider deliberating whether to issue an overdose of medication to relieve the suffering of a patient following a request by the patient to do so.
V. Students in university campaigning against an overseas policy.

The test uses a Likert-type scale for respondents to rate and rank responses to the dilemmas. They are invited to rate 12 statements with respect to each dilemma followed by ranking of what they consider to be the four most important. The statements do not comprise conclusive positions regarding the dilemma but are presented as elements of reasoning to which respondents must determine meaning. The DIT-2 questionnaire also required respondents to provide additional demographic data including information related to their political orientation. This required the students to describe themselves as being one of the following: ‘very conservative’, ‘somewhat conservative’, ‘neither conservative nor liberal’, ‘somewhat liberal’ or ‘very liberal’. The self-described political responses were converted to numerical values: [‘very conservative’: 5; ‘somewhat conservative’: 4; ‘neither conservative nor liberal’: 3; ‘somewhat liberal’: 2 and ‘very liberal’: 1] to enable statistical evaluation. However, all other demographic questions including those enquiring whether respondents were citizens of the USA and level of education, age and gender were omitted from the DIT-2 questionnaire to eliminate duplication of questions and minimise the duration of time to undertake the survey.

The DIT-2 is ‘owned’ and copyrighted by the University of Alabama, and a condition of its use included limiting the amount of change that could be made to it [21]. However, the inclusion of two undergraduate final-year BDS students in the pre-piloting and piloting process ensured its acceptability. The time taken to complete the survey was approximately 25 min.

### 2.2. Survey Respondents

Potential respondents were 66 final-year BDS students who were within one month of the completion of their dentistry studies at the UofA. The final-year BDS coordinator informed the students about the presence of the survey within the university’s student software platform on 28 October 2024, where a cover letter, information regarding, and a link to, the survey could be accessed. The students were reminded of the presence of the survey on 18 November, with 30 November being the last day that students could respond before the survey was closed. On completion of the survey, students were provided with the opportunity to enter a prize draw for AU$250. It was not possible to link prize draw entries with the identity of the respondents of the survey. The responses from the DIT-2 were imported into a Microsoft (Redmond, WA, USA) Excel spreadsheet and sent for scoring to the University of Alabama. The responses from Section 1 were also imported into a Microsoft Excel spreadsheet where the data were arranged in such a manner as to enable alignment with the results from the University of Alabama.

### 2.3. Statistical Analysis

Statistical analyses were carried out using SPSS, version 27 (IBM, Armonk, NY, USA). The Shapiro–Wilk test was used to ascertain whether the data were distributed normally. Where data were distributed normally, means and 95% confidence intervals (CIs) were calculated. Medians and interquartile (IQR) values were provided for non-normally distributed data. *T*-tests were used to determine whether there were statistical differences between groups with normally distributed data, and Mann–Whitney tests were utilised to identify statistically significant differences between non-normally distributed data.

The Spearman rank correlation coefficient test was then employed to determine whether there was an association between respondents’ self-described political orientation and the four moral reasoning schema scores—P, PI, MN and N2. The test was also used to investigate whether there was an association between respondents’ age and the four schemata. The level of association was considered to be very strong if r values were between ±0.90 to ±1; strong if r values were between ±0.70 and ±0.89; moderate if values were between ±0.40 and ±0.69; weak if values were between ±0.10 and ±0.39; and minimal for values between ±0.0 and 0.09 [41].

## 3. Results

Following exclusion of incomplete surveys from four (6.1%) respondents, a response rate of 45.5% (n = 30) comprising 16 (53.3%) females and 14 males (46.7%) was documented. The median (IQR) age of the sample was 23.0 (23.0, 23.5) years. There was no difference (*p*= 0.66) in the median (IQR) age of female [23.0 (23.0, 26.0)] and male [23.0 (23.0, 35.0)] respondents. Based on Yamane’s Formula, this meant that there was a 95% certainty that the responses were within a 13% margin of error.

Twenty-two (72.3%) respondents reported that English was their first language. Twenty-three (76.7%) responded that they had received the majority of their pre-BDS education in Australia or New Zealand, with seven (27.7%) reporting that most of their pre-BDS education was elsewhere.

Table 1 shows that 60% of the sample described themselves as being neither conservative nor liberal.

There was no difference (*p* = 0.83) in self-described political orientation between female (median (IQR): 3 (3.0, 3.0)) and male (median (IQR): 3 (2.75, 3.25)) respondents.

Table 2 shows the mean (95% CI) scores for the four schemata of the DIT-2 tool. The mean (95% CI) N2 score was 42.12 (37.72, 46.53). There were no significant differences (*p* > 0.05) for any of the schemata between female and male respondents.

Table 3 shows that there was no statistically significant difference in the four schemata scores between those who had received the majority of their pre-BDS education in Australia/New Zealand and those whose who received it elsewhere.

Table 4 shows that there was no difference in the scores between those whose first language was English and those where English was not their first language.

Table 5 shows that age was moderately associated with N2 scores.

## 4. Discussion

This is the first study to investigate the levels of moral reasoning among undergraduate dentistry students in Australia.

The mean (95% CI) P and N2 scores were 37.80 (32.04, 43.56) and 42.12 (37.72, 46.53), respectively. The findings indicated that the scores for females and for those who had undertaken the majority of their pre-BDS studies outside of Australia and New Zealand were higher, but the differences were not statistically significant. That patients regularly present clinicians with an ethical dilemma or problem emphasises the present study’s relevance [42]. However, although the P and N2 scores were similar to studies among dental students conducted in other countries, they were less than the scores considered optimal for a healthcare provider to proficiently manage challenges to ethical decision-making.

The number of studies evaluating moral reasoning among undergraduate dentistry students are few, and the number utilising the DIT-2 tool is fewer still. Although Rest suggested that a P score greater than 50 indicated moral reasoning at optimal levels, what constitutes acceptable mean P and N2 scores among tested cohorts has not been definitively established [43]. A recent study by Gungordu et al. [16], however, has provided normative information related to over 73,000 tested individuals in investigations published between 2011 and 2020. In addition, comparison with other undergraduate clinical healthcare disciplines using the DIT and DIT-2 tools can provide additional context to the findings reported here. The mean P and N2 scores in the present survey were 37.8 and 42.1, respectively. This corresponded to the mean P and N2 scores of 36.1 and 35.7 in Gungordu et al.’s assessment of data related to individuals of similar age and educational background [16]. The P score in this study compared with P scores of 34.3 to 38.0 among first-year undergraduate dentistry students in the US using the DIT-2 [22]. It also compared with P scores of 36.3 to 40.3 among ‘all’ undergraduate dentistry students in a Brazilian dental school and 42.3 to 52.9 among pre-doctoral dentistry students in a US university using the DIT [35,37]. However, it was greater than the mean P score found in final-year undergraduate pharmacy students in the UK and was slightly higher than the 32.3 to 35.2 scores observed among medical students in Croatia [20,27]. By contrast, P scores in the present study were less than the 43.6 to 49.0 reported in a study among nurses in Iran and 51.9 in a study investigating moral reasoning in orthopaedic surgery residents [18,32].

Why there should be variance in scores between students in different clinical environments is unclear. The nature of the environment in which nursing and postgraduate medical students carry out clinical activity may have enabled more immediate confrontation with issues that required deeper ethical consideration and resulted in the development of more sophisticated moral reasoning skills [18,20,28]. In addition, the need to adhere to regulations, protocols and rules of controlled educational and clinical environments may have hindered the development of moral reasoning skills among undergraduate dental and pharmacy students [27,28,44].

The P and N2 scores were higher among females in the present study. However, the differences did not reach significance. This replicated the findings to the study among dentistry students in the US [22,37]. Although controversy surrounds the role of sex in moral reasoning [45], the analysis by Gungordu et al. [16] suggests that DIT-2 P and N2 scores show minimal differences between males and females at lower educational levels, but the differences tend to become more prominent as respondent level of education increases.

The present study found that increasing age was moderately associated with N2 scores. This appears to be a consistent finding among young adults (the demographic of the present study) in the literature [15,16,46], with a decline in moral reasoning being recorded in older adults by some researchers [27,47,48]. Lee et al. speculated that this may be a result of the exploration of behaviours by young people in their twenties who are seeking to formulate their moral identities—a journey initiated in adolescence and continually being developed and refined into young adulthood [46].

Formal training in ethics during their BDS studies at the UofA is limited to four hours in the pre-clinical first year. Research, however, has indicated that the introduction of relevant education during their clinical learning may increase moral reasoning scores. Activities that have resulted in improved moral reasoning performance include involvement in programmes focusing on equality, diversity and inclusion [49], modules that concentrate on morally specific themes [50] or promote ethical problem-solving [51], and extra-curricular involvement in service learning [52]. Additional approaches employed to optimise or increase moral reasoning scores among students undergoing clinical education include case-based learning [32,34], multiple retakes of the DIT-2 during undergraduate programmes [53] and simulation-based teaching [33]. Care, however, is required to ensure that the appropriate mode of teaching in ethics is chosen, as not all interventions [30] necessarily improve moral reasoning scores among students.

The shortcomings of the present investigation require acknowledgement. The study was limited to one university in Australia, so the findings should be applied more generally with care. The survey response rate was disappointing and reflected the general reduction in response rates to surveys observed in recent years and the challenges in obtaining high numbers of respondents in surveys to which it takes approximately 25 min to complete. Future research should consider on how to maximise response rates in surveys such as these to ensure greater confidence in the conclusions made from participant responses. Nevertheless, the response rate was higher than the average response rate of 44.1% in 1071 online surveys in education-related research calculated in a 2022 meta-analysis [54] and provides baseline data for future similar investigations in Australia and elsewhere. Moreover, the cross-sectional nature of the study means that the findings relate to a ‘snapshot’ in time. Future research should involve the incorporation of longitudinal data acquisition to determine the dynamic process of the development of moral reasoning. This, in turn, will potentially enable evaluation of changing moral reasoning scores and behaviour as undergraduate dentistry students transition into independent dental practice. In addition, it is important to note that the measurement values recorded in the present investigation do not suggest a particular standard of achieved morality but rather suggest the reasoning that underpins the selection of behaviours in challenging circumstances that necessitate moral reasoning [55]. Furthermore, a purported shortcoming of Kohlberg’s theory [24], from which the DIT and DIT-2 is derived, is the assumption that a concentration on relationships was indicative of deficiency in moral reasoning development. Prescott et al. contended, that in this scenario, high scores at the MN level may be indicative of the acquisition of skills essential for the provision of patient-centred and patient-focused care [28].

A strength of the study, however, was the utility of a validated psychological instrument that has high test–retest reliability [21] and satisfactory internal consistency [19] and which has been used widely among undergraduate pharmacy, medical, law, veterinary, nursing and dentistry students [18]. It is the first study of its kind to be conducted in the dental domain in Australia. The findings will enable reflection on the teaching of ethics to dentistry students within the UofA and dental schools more widely. In addition, it provides baseline information for future research regarding moral reasoning and ethical conduct related to dentistry students and dentists in practice.

## 5. Conclusions

The present study used a validated moral reasoning instrument to determine the moral reasoning of final-year undergraduate BDS students in Australia and provided baseline data for future pertinent investigations. The moral reasoning scores were comparable to studies among similar cohorts conducted in other countries but were less than the scores considered by Kohlberg to be optimal for a healthcare provider who is confronted with ethical decision-making challenges. Moral reasoning scores were higher among females and among those who received most of their pre-BDS education outside of Australia and New Zealand, but the differences were not significant. There was a moderate association between age and N2 scores. Consideration should be given to the introduction of appropriate formal training in ethics to potentially assist in dental student management of ethical challenges.

## Figures and Tables

**Table 1:** Respondent description of political orientation (N = 30).

Political Orientation	N	%
Very conservative	1	3.3
Somewhat conservative	5	16.7
Neither conservative nor liberal	18	60.0
Somewhat liberal	4	13.3
Very liberal	2	6.7

Key. N: number; %: percentage.

**Table 2:** Mean (95% CI) P, PI, MN and N2 scores.

DIT-2			Sex				
	Total		Male		Female		* *p*
	Mean	95% CI	Mean	95% CI	Mean	95% CI	
P	37.80	32.04, 43.56	33.00	24.06, 41.90	42.00	34.25, 49.75	0.11
PI	18.33	14.47, 22.2	18.43	12.28, 24.58	18.25	12.69, 23.81	0.93
MN	39.07	32.98, 45.15	43.29	34.22, 52.35	35.38	26.64, 44.11	0.19
N2	42.12	37.72, 46.53	39.08	32.2, 45.97	44.78	38.71, 50.85	0.12

Key. CI: confidence interval. * *p* < 0.05 indicates statistical significance. P: Postconventional. PI: Personal Interest. MN: Maintaining Norms. DIT: Defining Issues Test.

**Table 3:** Moral reasoning scores according to where the majority of pre-BDS education was undertaken (N = 30).

	Pre-BDS Education				
Schemata	Aus/Non-Aus/NZ (N = 23)		Non-Aus/NZ (N = 7)		
	Mean	95% CI	Mean	95% CI	* *p*
P	36.43	29.92, 42.95	42.29	26.65, 57.92	0.39
PI	19.30	15.12, 23.49	15.14	3.5, 26.79	0.36
MN	39.91	32.79, 47.04	36.29	20.91, 51.67	0.61
N2	41.60	36.9, 46.30	43.84	29.67, 58.02	0.67

Key. BDS: Bachelor of Dental Surgery. N = number. Aus: Australia. NZ: New Zealand. CI: confidence interval. * *p* < 0.05 indicates statistical significance. P: Postconventional. PI: Personal Interest MN: Maintaining Norms.

**Table 4:** Moral reasoning scores according to respondents’ first language (N = 30).

	1st Language				
Schemata	Eng (N = 22)		Non-Eng (N = 8)		
	Mean	95% CI	Mean	95% CI	* *p*
P	37.00	29.75, 44.25	40.00	28.91, 51.09	0.65
PI	20.00	15.15, 24.85	13.75	7.73, 19.77	0.15
MN	37.27	29.71, 44.83	44.0	32.48, 55.52	0.33
N2	40.56	34.92, 46.20	46.43	39.83, 53.02	0.23

Key. N = number. 1st: First. Eng: English.: Australia. NZ: New Zealand. CI: confidence interval. * *p* < 0.05 indicates statistical significance. P: Postconventional. PI: Personal Interest MN: Maintaining Norms.

**Table 5:** Association of age and self-described political orientation with moral reasoning scores.

	Schemata											
	P			PI			MN			N2		
	r	95% CI	* *p*	r	95% CI	* *p*	r	95% CI	* *p*	r	95% CI	* *p*
Age	0.36	−0.03, 0.63	0.06	−0.16	−0.50, 0.23	0.41	−0.33	−0.62, 0.05	0.08	0.41	0.01, 0.65	0.04
Pol Or	−0.18	−0.52 to 0.20	0.34	0.02	−0.35, 0.39	0.92	0.22	−0.17, 0.54	0.26	−0.28	−0.59, 0.10	0.14

Key: P: Postconventional. PI: Personal Interest MN: Maintaining Norms. r: Rho. CI: confidence interval. * *p* < 0.05 indicates statistical significance. Pol Or: political orientation.

## Data Availability

The original contributions presented in this study are included in the article. Further inquiries can be directed to the corresponding author.

## Abbreviations

The following abbreviations are used in this manuscript:

UofA	University of Adelaide
BDS	Bachelor of Dental Surgery
DIT	Defining Issues Test
CI	Confidence Interval
MN	Maintaining Norms
STROBE	Strengthening the Reporting of Observational Studies in Epidemiology
IQR	Interquartile
